# Democracy, transparency, economic development, religion, and women on boards of national Olympic committees: Evidence from 89 countries

**DOI:** 10.1371/journal.pone.0342030

**Published:** 2026-02-25

**Authors:** Yeayoung Noh, Na Young Ahn, Seungmin Kang

**Affiliations:** 1 Department of Sport Science, Kongju National University, Gongju, South Korea; 2 Faculty of Business and Law, Bournemouth University, Poole, United Kingdom; 3 Department of Health, Nutrition, and Exercise Sciences, North Dakota State University, Fargo, North Dakota, United States of America; Sri Lanka Institute of Information Technology - Malabe Campus: Sri Lanka Institute of Information Technology, SRI LANKA

## Abstract

The authors investigate the connection between a country’s level of democracy, transparency, economic development, religion, and the representation of women on the national Olympic committees’ (NOC) boards. Using data from 89 countries, they analysed how democracy, governance quality, gross national income per capita, and religious composition are associated with women’s representation on NOC boards. Results reveal that countries with higher levels of democracy, greater transparency scores, higher economic development, a large proportion of Protestants, and a higher proportion of Jewish adherents were related to a greater proportion of women on NOC boards. The findings highlight the importance of democratic institutions, governance quality, and economic prosperity in creating conditions that support women’s advancement into decision-making roles, while religious context further influences cultural and normative factors that influence gender equality. Further research should explore the interplay among types of democracy, religious indices, and geographical characteristics to better understand the mechanism driving gender-balanced leadership. Overall, promoting democratic principles, participatory and inclusive governance, transparency, economic opportunity, and religious freedom can facilitate inclusive societal norms and enhance gender diversity, equity, and inclusion leadership roles.

## Introduction

Despite growing efforts to promote gender balance in leadership, women remain underrepresented in corporate and governance board positions worldwide, holding less than 25% of these roles [1,2]. The landscape of leadership is influenced by gender, with men still predominantly occupying senior leadership positions. However, the prevalence of women in leadership varies across countries and regions. For instance, the percentage of women legislators ranges from 16.7% in the Pacific nations to 31.7% in Europe [3]. In the corporate sphere, women hold only 19.7% of board positions, 10.8% of board chair roles, and 16.3% of chief executive positions [4]. These disparities raise questions about the factors contributing to the variations in gender inequality in leadership across countries and regions. Understanding the nuances behind these disparities is critical for developing strategies aimed at enhancing gender equity in leadership roles.

To address this issue, researchers have examined various barriers at different levels to explain the underrepresentation of women in leadership positions. These include individual-level factors, such as human and social capital, as well as group-level factors, such as social identity, network, and in-group bias [5]. At the organisational level, work-life balance and career interruptions have been identified as barriers to women’s advancement [6]. Societal-level factors, such as gender role stereotypes and ideologies also hinder women’s progress into leadership [7]. For example, one prominent framework that illustrates this phenomenon is role congruity theory, which suggests that women leaders are perceived as assertive and less favourable than men due to traditional gender beliefs, a trait associated with gender bias in leadership evaluation [8]. This misalignment triggers gender norms in the evaluation of leadership capabilities, perpetuating inequality in access to leadership positions. Given the prevalence of gender bias in leadership globally and the role of societal norms and stereotypes in shaping these biases, we focus on the societal-level factors in this study. Exploring how societal attitudes and cultural narratives shape perceptions of women’s leadership informs strategies to tackle stereotypes and promote a more equitable environment for aspiring women leaders.

This approach underscores the importance of representation: from a critical mass perspective, having a minimum of three women on boards can result in more innovative decision-making, improved policy outcomes, and a more supportive workplace environment [7]. By prioritising this representation, we can overcome the limitation of tokenism, as policymakers and organisations have prioritised reducing the gender gap in leadership [9]. Identifying the characteristics of countries that can reduce gender imbalance in leadership can guide us towards a more inclusive and equitable leadership composition. Moreover, researchers have shown that women on the boards can have a positive impact on both organisations and society, promoting gender equality and social progress [10]. The benefits of achieving gender balance for businesses and organisations include ethical decision-making [11], enhanced performance [12] and access to a broader talent pool [6].

In this study, we investigate the effects of democracy, transparency, economic development, and religion on gender equality in leadership. Democracy is defined as a regime that prioritises free voting, fair and equal distribution of resources and power, the rights of opposition parties and minorities, and freedom of expression [13]. This concept suggests that democratic countries are more likely to foster gender balance in leadership and encourage participation from both women and men in policymaking processes [14]. However, empirical evidence shows inconclusive results. For example, some researchers have found a positive link between democracy and increased women’s representation in parliament worldwide [15]. In contrast, others reported that authoritarian regimes can also lead to high rates of women in parliament, as seen in Cuba and Rwanda [16]. As a result, the extent to which the level of democracy is associated with greater representation of women in leadership positions remains unclear.

Beyond democratic institutions, the quality of governance and a country’s level of economic development further impact opportunities for women’s leadership [17]. Higher levels of transparency and lower corruption are associated with stronger accountability, merit-based selection processes, and more inclusive institutional environments, which can facilitate women’s access to decision-making positions [18,19]. Similarly, economic development is often linked to expanded educational opportunities, labour force participation, and shifting social norms regarding gender roles, all of which may support women’s advancement into leadership [20]. However, prior researchers have suggested that transparent governance and economic prosperity, while important, do not automatically lead to gender-balanced leadership, as their effects are conditional and context-dependent.

In addition to democracy and governance conditions, religion is another factor that influences gender equality on NOC boards. While religion provides a sense of meaning and purpose to individuals and communities, its impact on economic development and policymaking has been controversial [21]. A causal link between a particular religion and women’s representation in leadership positions is also contentious. Some evidence indicates greater women’s visibility in leadership in Protestant-majority contexts, while others highlight more hierarchical gender ideologies in certain religious traditions (e.g., Catholicism, Confucianism, and Islam) that may pose challenges to women’s representation [22–25]. However, these patterns are not universal and often depend on historical and cultural factors. For instance, Lam [23] pointed to the patriarchal aspects of Muslim and Catholic traditions, which could contribute to lower representation of women in leadership. Moreover, the growing population of religiously unaffiliated individuals (agnostics and atheists) tends to support liberal political issues and greater women’s representation in top positions [26–28]. Overall, empirical evidence is inconclusive, underscoring the complex and conditional relationships among democracy, transparency, economic development, religion, and women’s representation on NOC boards.

Building on the existing body of research on the underrepresentation of women in leadership, we shift our focus to the macro-level factors—including democracy, transparent governance, economic development, and religion to examine how they collectively shape women’s representation on NOC boards. While most of the work on gender equality has been conducted at a single country level [7,29,30], notable exceptions have adopted a comparative approach [4,31]. Our cross-national comparison expands on this scholarship by assessing how political, institutional, economic, and cultural conditions interact to influence women’s leadership representation across countries.

## Conceptual framework

### Democracy, transparency, economic development, and women’s representation in leadership

Schumpeter [32, p. 269] defined democracy as an “institutional arrangement for arriving at political decisions in which individuals acquire the power to decide by means of a competitive struggle for the people’s vote.” However, this view is limited to procedural aspects of liberal democracy and may not fully capture the complexity of democratic systems observed around the world today. In reality, democracy is recognised as a system that extends beyond electoral competition to encompass a broader range of features, including civic democracy, which includes freedom, rule of law, accountability, equal rights and opportunities, participation, competition, responsiveness, transparency, and effective representation [13]. Collectively, these multiple aspects reflect a more civic and substantive understanding of democracy, where fair distribution of power and resources and the alignment between social and political leaders and the public are centrally valued.

As such, the presence of women in decision-making positions in social and political spheres is regarded as a critical factor for a country’s democratic quality. The dearth of women in such positions is often a result of democratic deficits [14,16]. From a critical mass theory perspective, when at least three women are present in decision-making roles, their presence can lead to meaningful influence by promoting not just visibility but also active participation and impact on strategic directions and board discussions [33]. However, this connection varies greatly across countries and political systems [24]. For example, this variation is evident in some non-democratic or hybrid regimes like Rwanda, Cuba, and Bolivia, which hold a high percentage of women in national parliaments despite authoritarian or moderately democratic regimes [3]. Moreover, monarchical countries like Nepal and the United Arab Emirates have more than 30% of women in political leadership positions. In contrast, fully democratic countries, such as Uruguay, Ireland, Malta, and the Republic of Korea show lower levels of women’s representation in leadership [34]. Given that nearly half of the world’s countries (167; 45.5%) are classified as democratic [35], these divergent patterns raise questions of which type of governmental system is more conducive to enhancing women’s representation in leadership.

While some researchers have argued that the quality of democracy in a country is not necessarily linked to greater representation of women in decision-making positions [36], others have found that authoritarian or monarchic regimes can actually have more women in government [24]. For example, monarchic countries held more women in national government positions [37]. However, this phenomenon may be driven by different dynamics of power and representation. While authoritarian regimes could place more women in leadership roles, these systems often lack fundamental democratic features, such as civil liberties, free and fair elections, and accountability. As Tremblay [16] pointed out, this means that the relationship between democracy and gender balance in leadership is not straightforward and may, in fact, be weak or even non-existent. This complexity suggests that simply increasing the number of women in leadership positions does not inherently equate to improved governance or democratic health. Consequently, the quality of the political systems in which these women are placed highlights the importance of evaluating both the quantity of representation and the contextual factors that influence the effectiveness and impact of women leaders. This challenges the assumption that representation alone is sufficient to drive progress in gender equality.

Contrary to these findings, some researchers have supported the positive linkage between democracy and a high rate of women in leadership, offering a more optimistic view from a comparative, cross-national perspective [15]. Inglehart and colleagues [20] examined whether a high level of democracy is related to greater women’s representation in political leadership. Using the World Values Surveys (WVS), they found that countries with higher democratic governance tend to correspond with a higher rate of women in parliament and more egalitarian values towards gender equality and self-expression. Likewise, Esarey and Schwindt-Bayer [38] demonstrated that democracy significantly influenced both the presence of women in political leadership and lower levels of corruption in a country. Together, using the Worldwide Governance Indicators, Hao and colleagues [39] found that having more women on corporate boards reduced corruption, while democracy promoted the value of gender equality on boards. Based on these cross-cultural reviews, the level of democracy is a robust indicator of gender balance in positions of power within a nation [40], which leads to Hypothesis 1a:

**Hypothesis (H1a):** The democratic regime of a country will be positively associated with a greater proportion of women on NOC boards.

Beyond the democratic features of a political system, the quality of governance plays a critical role in shaping the presence and advancement of women in decision-making positions [17]. Researchers have demonstrated that good governance, characterised by lower corruption and higher transparency, is linked to greater accountability in public institutions and organisational boards, creating an environment more conducive to gender equality in leadership [18,19,38]. Together, these studies contribute to an ongoing discussion about the relationship between gender and corruption, suggesting that good institutions promote both more transparent governance and better gender equality. By considering governance alongside democratic measures, we account for both the political and institutional contexts that facilitate or hinder women’s representation in leadership positions. We, therefore, propose the following:

**Hypothesis (H1b)**: Higher levels of transparency of a country will be positively associated with a greater proportion of women on NOC boards.

In addition to political institutions, a country’s level of economic development also influences women’s access to leadership roles. Countries with higher levels of economic development tend to offer greater educational and employment opportunities for women and foster social environments that are supportive of gender equality in leadership positions [17]. Nonetheless, economic development alone is unlikely to ensure equal representation in leadership, as its influence may depend on broader institutional and cultural contexts [20]. Based on this perspective, we posit the following:

**Hypothesis (H1c)**: Higher levels of economic development of a country will be positively associated with a greater proportion of women on NOC boards.

### Religion and women’s representation in leadership

Huntington [41] contended that religion plays a crucial role in shaping a country’s national identity and ideology by drawing on its religious traditions and cultural heritage. Researchers have highlighted the significance of religious beliefs and practices in providing individuals and communities with a sense of purpose and meaning, and influencing politics, social norms, civic behaviours, and gender roles [14,42,43]. Additionally, researchers have explored how religion relates to other important aspects of society, including economic growth [44], social capital [45], or political landscape [46]. Relevant to our study, others have examined the impact of religion on gender inequality issues in leadership domains [15]. Despite such efforts, conceptualising a clear causal mechanism between religion and gender balance in leadership is challenging due to the complex and multilateral nature of religion [21]. To better understand this relationship, researchers have used cross-national studies to investigate the interplay of religion with socioeconomic and political outcomes [23].

Specifically, researchers have explored the connection between long-standing religious institutions and women’s representation in leadership globally. Empirical evidence from cross-national research illustrates several recurring patterns. First, countries with hierarchical religious institutions (e.g., Catholic, Muslim, or Confucian) lean towards fewer shares of women in positions of power [46]. This could be due to the patriarchal, elitist, and hierarchical nature of these institutions, which may perpetuate gender inequality. Conversely, countries with a large percentage of Protestants have greater gender balance in leadership positions [20,46]. This trend may be attributed to the differing emphasis on hierarchy: although both Catholicism and Protestantism are branches of Christianity, Protestantism places less rigid emphasis on hierarchical order and has more egalitarian views on gender, which could create a more supportive environment for women’s participation in decision-making roles [47]. Finally, in countries where there are high shares of the population belonging to Hindu, Buddhist, or Jewish denominations showed mixed trends. Reynolds [24] found a weak negative association between each of the three religious denominations and a high share of women in legislatures, as did Kenworthy and Malami [22]. However, this contradicted the findings of Paxton [36], suggesting that Hinduism and Judaism were not linked with the representation of women in leadership. Overall, the evidence suggests that a higher percentage of the population belonging to the Protestant denomination is more likely to support women’s entry into leadership roles than non-Protestant denominations, such as Catholic, Muslim, Buddhist, Jewish, and Hindu. Synthesising these insights, we expect a national religious context, particularly the predominance of Protestant versus more hierarchical faiths, to meaningfully influence the proportion of women in leadership positions, such as those on NOC boards. This leads to Hypothesis 2 and Hypothesis 3a-e:

**Hypothesis (H2):** The percentage of the population following a Protestant religious tradition will be positively associated with a greater proportion of women on NOC boards.

**Hypothesis (H3):** The percentage of the population following Buddhist (H3a), Catholic (H3b), Hindu (H3c), Jewish (H3d), and Muslim (H3e) religious traditions respectively, will be negatively associated with a greater proportion of women on NOC boards.

Recently, researchers have focused on the growing trend of religiously unaffiliated demographic across countries. According to Pew Research Center [27], approximately 16% of the global population does not identify with a religion, and this population is growing globally. There is some evidence suggesting that these religiously unaffiliated individuals are more likely to hold liberal views on issues, such as abortion, contraception, divorce, and same-sex marriage because they consider themselves to be egalitarian and have more progressive political attitudes compared to those religious adherents [26].

Moreover, historically dominant religious institutions often embrace socially and politically conservative views on gender roles and are typically led by men [48]. This dynamic might result in individuals distancing themselves from their religion, and fewer women holding top positions within such religious organisations [47]. This distancing from traditional religious structures may empower individuals to seek out alternative forms of community and leadership that fit closely with their personal values and beliefs.

There is further cross-cultural evidence supporting this conjecture. Schnable’s [28] analysis of large-scale data from the ARDA found that countries with a high percentage of the unaffiliated demographic were positively associated with greater representation of women on boards. For instance, countries like Sweden and Denmark, which boast high levels of religious disaffiliation exhibit significant gender balance in corporate leadership roles. Given this finding, one might expect that the countries where the non-religious population is prominent would have greater gender equality on boards. This leads to Hypothesis 4:

**Hypothesis (H4):** The percentage of the population that is religiously unaffiliated will be positively associated with a greater proportion of women on NOC boards.

## Materials and methods

### Data source

We employed multiple secondary datasets. First, we gathered data from the official website of the 2016 Summer Olympics and the IOC. We collected the total number of athletes, the number of board members, and their genders. We then accessed these webpages of each NOC (https://www.olympic.org/national-olympic-committees), where we obtained profiles of their boards of directors. Second, we compiled a comprehensive democracy index from 167 countries over a 5-year period (2005–2010). This dataset is publicly available through the EIU database (http://www.eiu.com/public/thankyou_download.aspx?activity=download&campaignid=democracyindex2019) and is widely used to measure democracy [49]. Furthermore, we incorporated a transparency index from 178 countries for the year 2010 to account for cross-national variation in perceived public sector corruption (https://www.transparency.org/en/cpi/2010). We also utilised the world religion dataset as another country-level indicator, which was derived from the ARDA and included data from 188 countries for the year 2005 and 2010 [50]; http://www.thearda.com/Archive/Files/Descriptions/WRPGLOBL.asp]. This publicly available dataset includes information on the number and share of adherents from various religions and the religiously unaffiliated population for each country. Finally, we included the World Bank’s gross national income (GNI) per capita data for the year 2010, a widely recognised indicator of economic development (https://data.worldbank.org/indicator/NY.GNP.PCAP.CD). The descriptive statistics and variables used in the analysis are presented in Table 1.

**Table 1:** An overview of variables and descriptive statistics.

Variables	Description	*N*	*M*	*SD*	Min.	Max.
% women NOC members	Percentage of women NOC members	89	.198	.118	0	.500
Logged NOC members	Total number of NOC members	89	1.124	.197	.698	1.579
No woman	No women on NOC boards	89	.078	---	0	1
1 woman	One woman on NOC boards	89	.213	---	0	1
2 women	2 women on NOC boards	89	.202	---	0	1
3 and more women	3 and more women on NOC boards	89	.505	---	0	1
2016 Olympians	Total number of 2016 Olympians	89	106.516	127.147	2	567
Logged population size	Total population in each country	89	7.143	.701	5.368	9.122
Americas	Dummy for continent (1 = yes, 0 = otherwise)	89	.191	---	0	1
Asia	Dummy for continent (1 = yes, 0 = otherwise)	89	.134	---	0	1
Europe	Dummy for continent (1 = yes, 0 = otherwise)	89	.426	---	0	1
MENA	Dummy for continent (1 = yes, 0 = otherwise)	89	.078	---	0	1
Pacific	Dummy for continent (1 = yes, 0 = otherwise)	89	.044	---	0	1
sub-Saharan Africa	Dummy for continent (1 = yes, 0 = otherwise)	89	.123	---	0	1
Democracy	Overall democracy index, scores on a 0–10 scale	89	6.655	2.190	2.347	9.837
Transparency	Overall corruption index, scores on a 0–100 scale	88	4.637	2.311	1.4	9.3
Logged GNI per capita	Gross national income per capita	89	3.912	.592	2.724	4.949
Buddhism	Percentage of Buddhist adherents	89	.027	.114	0	.870
Catholicism	Percentage of Catholic adherents	89	.318	.327	0	.889
Hinduism	Percentage of Hinduism adherents	89	.029	.126	0	.808
Islam	Percentage of Muslim adherents	89	.180	.326	0	.990
Judaism	Percentage of Jewish adherents	89	.001	.002	0	.018
Protestantism	Percentage of Protestant adherents	89	.148	.212	0	.871
Religious Adherents	Percentage of all religious adherents	89	.986	.019	.911	.999
Unaffiliated Population	Percentage of religiously unaffiliated population	89	.127	.166	0	.923

**Notes**: NOC = National Olympic Committee. *N* = the number of countries. *M* = mean. *SD* = standard deviation. Min = minimum. Max = maximum. MENA = Middle East and North Africa. GNI = the natural logarithm of a country’s gross national income per capita.

### Measures

#### Dependent variable.

The dependent variable is the proportion of women on NOC boards in 2016. The measure was calculated by dividing the number of women serving on each NOC board by the total number of NOC board members in each country, resulting in a scale ranging from 0 to 100, where 0 represents no women and 100 represents equal representation of women and men.

#### Independent variable.

The first independent variable, the EIU’s democracy index was selected for a number of reasons. First, the EIU’s index combines information from mass surveys and expert opinions [49]. Moreover, the EIU’s index is strongly correlated with other democracy indices, such as the Freedom in the World [51]. Finally, this index captures a broader concept of democracy, emphasising the overall democracy score for each country.

The EIU’s democratic criteria are based on four dimensions, which are combined to produce a score ranging from 0 to 10, with higher values indicating higher levels of democracy. To adapt to the current study, we used the average score of these four dimensions. We calculated the 5-year average of the democracy index for each country using the panel data.

We included another governance indicator: the transparency index, measured by the corruption perceptions index (CPI), to capture cross-national variation in perceived public sector corruption. Released annually by Transparency International (https://www.transparency.org/en/), this index ranges from 0 to 10, with higher scores indicating lower levels of corruption. This measure has been widely used in political science, economics, and business studies to assess governance quality, institutional integrity, and governance practices across countries [52]. For this study, we used the 2010 scores to align with the other country-level indicators.

Turning to the third independent variable, we employed the ARDA’s dataset on world religion. This dataset provides information on various religions across 233 countries. Particularly, we collected the cross-sectional data from six major religious denominations: Buddhist, Catholic, Hindu, Muslim, Jewish, and Protestant. We also calculated the percentage of adherents and of the religiously unaffiliated population for each composition. To compute these percentages (on a scale of 0–100), we divided the number of adherents of each religion by the total population of the country and multiplied by 100. For the percentage of the religiously unaffiliated population, we subtracted the number of unaffiliated individuals from the total number of religiously affiliated populations and divided by the total population. Since the data were observed in every 5-year interval, we estimated the average between 2005 and 2010.

Finally, GNI per capita served as an indicator of countries’ economic development. It is calculated as the total income of residents, including income from abroad, divided by the midyear population. In cross-national research, GNI per capita is often applied instead of gross domestic product (GDP) per capita because it more accurately reflects economic development and living standards of each country [53]. For this study, we took the natural logarithm of GNI per capita in current United States dollars to reduce skewness and improve comparability across countries, using the 2010 values to maintain consistency with the other indicators and minimise potential biases arising from differences in measurement years.

#### Control variables.

We included four control variables to account for potential confounding factors: the total number of athletes who competed in the 2016 Summer Olympic Games, NOC board size (natural logarithm), the number of women on NOC boards, and total population (natural logarithm). While the number of athletes and board members were normally distributed, total population was log-transformed due to skewness, and board size was also log-transformed to reduce scale differences across countries. To control for the number of women on NOC boards, we used dummy variables based on Wicker and colleagues’ categories [7]: No woman, 1 woman, 2 women, and 3 and more women. We also included six dummy variables to control for geographical region: the Americas (the baseline category), Asia, Europe, Middle East and North Africa, the Pacific, and sub-Saharan Africa. As Maoz and Henderson [50] elucidated, religion is closely tied to regional cultures, shaping similar or different patterns of religious diversity across regions. For example, Setzler [47] found that Catholicism is prevalent in most Latin countries, while Hinduism, Confucianism, or Islam are dominant in Middle East, South, and East Asia [54]. Similarly, Norris and Inglehart [15] mentioned that there is variation in the degree of democracy and presence of women in leadership across and within regions. We included six dummy variables for region to account for any spatial impact on the dependent variable, with a coding scheme of 1 for yes and 0 otherwise.

### Empirical analyses

Before conducting the regression analysis, we verified that all the necessary assumptions were met. We checked for multicollinearity, which was not an issue since all variance inflation factors were below the threshold of 10 [55]. Further, none of the other assumptions were violated; as such, we did not need to make further adjustments to the models or variables.

We specify the baseline estimating equation in which the proportion of women on NOC boards is expressed as a function of multiple country-level factors. Formally, the model is specified as: women_NOC = ƒ(democracy, transparency, religion, GNI, controls) + *ε*. The empirical regression model is then presented as follows: women_NOC_*i*_ = *β*_0_ + *β*_1_ democracy_*i*_ + *β*_2_ transparency_*i*_ + *β*_3_ religion_*i*_ + *β*_4_ln(GNI_*i*_) + *β*_5_ athletes_*i*_ + *β*_6_ln(boardsize_*i*_) + *β*_7_ women_dummy_*i*_ + *β*_8_ln(population_*i*_) + ∑ ^*β*^region + *ε*_*i*_.

We then used ordinary least squares (OLS) estimations to perform a set of regression analyses. First, Model 1 included all control variables: size of the Olympic movement in each country (the number of athletes and natural logarithm of board members), dummies for the number of women on the NOC boards, the log-transformed total population, and dummies for regions. Next, we estimated three models for each hypothesis testing. In Model 2, we added democracy index; in Model 3, we introduced transparency to indicate governance quality. Model 4 included economic development, measured by log-transformed GNI per capita. In Model 5, we added six religions; and in Model 6, we added the percentage of religiously unaffiliated populations. Finally, Model 7 estimated a full model that incorporated all independent variables. The same set of control variables was included in each model. We used robust standard errors for all regression models to account for potential heteroskedasticity and compositional differences in country-level variables [56,57]. We performed all statistical analyses using Stata software (version 19.5; StataCorp, College Station, the United States of America [USA]).

## Results

### Descriptive statistics

The bivariate correlation matrix is presented in Table 2. The average proportion of women on NOC boards was 19.8% (*SD* = .11), with an average board size of 14.7 members. The average number of athletes who competed in the 2016 Summer Olympics was 106.51 (*SD* = 127.14), and the average democracy index score was 6.65 (*SD* = 2.19), indicating that most countries have a mixed political system. The mean transparency index score was 4.63 (*SD* = 2.31), suggesting substantial cross-national variation in perceived governance quality. The average logged GNI per capita was 3.91 (*SD* = .59), reflecting wide disparities in levels of economic development across countries. In terms of religious affiliations, on average, there were 31.8% of Catholic, 18% of Muslim, 14.8% of Protestant, 2.9% of Hindu, 2.7% of Buddhist, and 0.1% of Jewish adherents. Of the total population in the ARDA dataset, 98.6% of those adhered to a particular religious denomination (*SD* = .01), while 1.2% had no religious affiliation (*SD* = .12).

**Table 2:** Bivariate correlation coefficients matrix.

Variables	1	2	3	4	5	6	7	8	9
1. % women NOC members	---								
2. Logged NOC members	−.099	---							
3. 2016 Olympians	.085	.155	---						
4. No woman	−.490**	−.129	−.102	---					
5. One woman	−.410**	−.260*	−.013	−.152	---				
6. Two women	−.072	−.110	−.094	−.147	−.262*	---			
7. Three or more women	.659**	.372**	.141	−.295**	−.526**	−.509**	---		
8. Logged population	−.206	.247*	.549**	.025	−.183	−.129	−.059	---	
9. Americas	−.007	−.142	.099	−.035	.095	.111	−.148	.101	---
10. Asia	−.202	.224*	−.099	.006	.195	−.116	−.070	.344**	−.191
11. Europe	.098	.069	.161	−.083	−.172	−.038	.217*	−.240*	−.419*
12. MENA	−.305**	.075	−.139	.224*	.153	.060	−.295**	.017	−.142
13. Pacific	.398**	−.132	.118	−.063	−.113	−.109	.214*	−.131	−.105
14. sub-Saharan Africa	.070	−.145	−.217*	.017	−.112	.065	.029	−.047	−.182
15. Democracy	.339**	−.050	.307**	−.170	−.133	−.032	.227*	−.147	.169
16. Transparency	.407**	−.012	.322**	−.065	−.196	−.057	.242*	−.251*	−.083
17. Logged GNI per capita	.252*	.081	.447**	−.024	−.223*	−.064	.248*	−.187	−.010
18. Buddhism	−.179	.065	−.045	.165	.104	−.111	−.084	.090	−.102
19. Catholicism	.035	−.139	.071	−.175	−.040	.149	.007	−.049	.431**
20. Hinduism	−.152	.168	−.108	−.005	.198	−.068	−.104	.099	−.080
21. Islam	−.247*	.110	−.235*	.111	.120	.039	−.189	.171	−.253*
22. Judaism	.173	.126	.604**	−.053	−.012	−.117	.133	.229*	.286**
23. Protestantism	.577**	−.262*	.003	−.157	−.105	−.004	.175	−.230**	.162
24. Religious Adherents	−.105	.032	−.366**	−.051	.138	.133	−.193	.051	.020
25. Unaffiliated Population	.103	−.027	.396**	.067	−.133	−.157	.199	.013	−.035
	10	11	12	13	14	15	16	17	18
10. Asia	---								
11. Europe	−.340**	---							
12. MENA	−.115	−.252*	---						
13. Pacific	−.085	−.187	−.063	---					
14. sub-Saharan Africa	−.148	−.324**	−.109	−.081	---				
15. Democracy	−.243*	.431**	−.415**	.076	−.305**	---			
16. Transparency	−.219*	.365**	−.103	.168	−.227*	.724**	---		
17. Logged GNI per capita	−.354**	.548**	−.053	.023	−.415**	.690**	.814**	---	
18. Buddhism	.538**	−.186	−.065	−.028	−.083	−.116	−.037	−.088	---
19. Catholicism	−.275**	.045	−.273**	−.082	−.022	.307**	.072	.184	−.192
20. Hinduism	.334**	−.215*	.005	.068	.032	−.045	−.084	−.220*	.010
21. Islam	.129	−.215*	.660**	−.101	.016	−.506**	−.279**	−.303**	−.076
22. Judaism	−.162	.018	−.119	.032	−.123	.292**	.328**	.351**	−.070
23. Protestantism	−.228*	.010	−.208	.191	.076	.324**	.363**	.201	−.122
24. Religious Adherents	.107	−.357*	.225*	−.086	.270*	−.424**	−.400**	−.464**	.040
25. Unaffiliated Population	−.081	.330*	−.219*	.108	−.258*	.394**	.384**	.438**	−.026
	19	20		21	22	23	24	25	
19. Catholicism	---								
20. Hinduism	−.184	---							
21. Islam	−.437**	−.004		---					
22. Judaism	.084	−.095		−.176	---				
23. Protestantism	−.112	−.094		−.320**	.079	---			
24. Religious Adherents	.015	.166		.369**	−.213*	−.063	---		
25. Unaffiliated Population	−.039	−.160		−.352**	.233*	.028	−.989**	---	

**Notes**: * *p* < .05. ** *p* < .01. MENA = Middle East and North Africa. GNI = the natural logarithm of a country’s gross national income per capita.

### Hypothesis testing

Model 1, with the control variables, accounted for 76.6% of the variance (*p* < .01). The addition of the democracy variable in Model 2 contributed an additional 1.4% of unique variance (*p* < .01). Consistent with H1a, democratic regimes were positively associated with a higher proportion of women on NOC boards (*b* = .013, *p* < .05). In Model 3, governance quality was introduced through the transparency index. The inclusion of transparency explained a further significant proportion of variance in women’s representation on NOC boards (Δ*R²* = .045, *p* < .01). Higher levels of transparency were positively associated with the proportion of women on NOC boards (*b* = .014, *p* < .01), providing support for H1b. Model 4 added economic development, measured by log-transformed GNI per capita, which also accounted for an additional 1.9% increase in explained variance. In line with H1c, higher levels of economic development were positively related to women’s representation on NOC boards (*b* = .046, *p* < .05).

We further predicted that countries with a high percentage of Protestants would be positively related to the proportion of women on NOC boards, whereas the percentage of Buddhist, Catholic, Hindu, Jewish, and Muslim adherents would have a negative association with the proportion of women on NOC boards. After adding six religions to test H2 and H3a–e, Model 5 explained an additional 10.4% of unique variance (*p* < .01). The results indicated that the percentage of Protestants was positively related to a higher proportion of women NOC members (*b* = .183, *p* < .01), supporting H2. Unexpectedly, the proportion of Jewish adherents was also positively associated with women’s representation on NOC boards (*b* = 9.183, *p* < .05), contrary to H3d. The remaining religious variables were not statistically significant, providing support for H3a–c and H3e.

We also hypothesised that a large percentage of the religiously unaffiliated population in a country would be positively related to gender balance on NOC boards. Although Model 6 and the associated change in *R*^*2*^ were statistically significant, the coefficient for the unaffiliated population was not, failing to support H4. Finally, we estimated the full model through a separate hierarchical multiple regression that included democracy, transparency, economic development, religion, and all control variables to examine their joint associations with the presence of women NOC members. Model 7 accounted for an additional 12.4% of unique variance (*p* < .01). The results showed that the share of Protestants and Jewish adherents remained positively related to gender equality on NOC boards (*b* = .148, *p* < .01; *b* = 7.666, *p* < .01, respectively), holding all others constant. Countries with higher transparency scores were positively associated with greater representation of women on NOC boards (*b* = .012, *p* < .01). Full results are presented in Table 3.

**Table 3:** Results of OLS models estimating the proportion of women on NOC boards (Listwise n = 89).

Variables	Proportion of Women on NOC boards									
	Model 1		Model 2		Model 3		Model 4		Model 5	
	*B*	*SE*	*B*	*SE*	*B*	*SE*	*B*	*SE*	*B*	*SE*
Constant	.569	.092	.459	.083	.326	.083	.269	.135	.348	.070
Logged NOC members	−.237	.047	−.232**	.045	−.224**	.037	−.236**	.042	−.202**	.041
No woman	−.308**	.029	−.303**	.028	−.302**	.021	−.308**	.026	−.282**	.027
One woman	−.206**	.015	−.203**	.014	−.193**	.013	−.194**	.015	−.190**	.013
Two women	−.115**	.015	−.112**	.014	−.106**	.013	−.107**	.014	−.096**	.012
Three and more women	REF		REF		REF		REF		REF	
2016 Olympians	.001	.001	−.001	.001	−.001*	.001	−.001	.001	−.001	.001
Logged population size	−.001	.013	.004	.013	.023	.012	.016	.014	.016	.011
Americas	REF		REF		REF		REF		REF	
Asia	−.008	.019	.003	.020	−.019	.017	−.001	.019	.023	.028
Europe	−.015	.018	−.016	.017	−.024	.015	−.025	.018	.017	.013
MENA	−.001	.019	.030	.023	−.007	.014	−.005	.018	.039	.034
Pacific	.089*	.037	.097**	.034	.104*	.051	.108*	.038	.108**	.028
sub-Saharan Africa	−.013	.022	.006	.025	−.006	.021	.009	.024	.003	.022
Democracy			.013*	.003						
Transparency					.014**	.003				
Logged GNI per capita							.046*	.017		
Buddhism									.006	.043
Catholicism									−.002	.024
Hinduism									−.009	.032
Islam									.001	.035
Judaism									9.183*	2.879
Protestantism									.183**	.028
Non-religion										
*R*^*2*^	.766		.780		.811		.785		.870	
Δ*R*^*2*^			.014*		.045**		.019**		.104**	
*F*	31.44**		35.89**		34.53**		30.87**		44.87**	
Variables	Proportion of Women on NOC boards									
	Model 6						Model 7			
	*B*		*SE*				*B*		*SE*	
Constant	.589		.099				.307		.116	
Logged NOC members	−.241**		.047				−.213**		.036	
No woman	−.306**		.029				−.288**		.021	
One woman	−.207**		.016				−.189**		.013	
Two women	−.117**		.015				−.096**		.011	
Three and more women	REF						REF			
2016 Olympians	.001		.001				−.001**		.001	
Logged population size	−.003		.014				.028*		.012	
Americas	REF						REF			
Asia	−.006		.020				−.001		.026	
Europe	−.014		.018				.003		.015	
MENA	−.001		.019				.002		.035	
Pacific	.089*		.037				.125**		.021	
sub-Saharan Africa	−.016		.022				−.016		.023	
Democracy							−.007		.005	
Transparency							.012**		.004	
Logged GNI per capita							.001		.021	
Buddhism							−.015		.045	
Catholicism							−.019		.025	
Hinduism							.016		.032	
Islam							−.014		.033	
Judaism							7.666**		2.717	
Protestantism							.148**		.036	
Non-religion	−.038		.042				−.048		.038	
*R* ^*2*^	.767**						.890**			
Δ*R*^*2*^	.001**						.124**			
*F*	30.15						63.78			

**Notes:** * *p* < .05. ** *p* < .01. MENA = Middle East and North Africa. GNI = the natural logarithm of a country’s gross national income per capita. REF = reference group. *B* = unstandardised regression coefficients. *SE* = standard error.

## Discussion

In this study, we examined the relationship between democracy, governance quality (transparency), economic development, religion, and women’s representation on NOC boards. The existing literature has raised questions about whether democratic countries consistently have more women in leadership [34] and has suggested that institutional transparency and economic prosperity may foster gender equality in leadership [18,19,38]. Further, some researchers have posited that dominant religions may influence the presence of women leaders in social and political spheres [22], while others have proposed that countries with a large proportion of religiously unaffiliated individuals exhibit gender balance in leadership [23]. Given mixed empirical evidence, we integrated these political, institutional, economic, and cultural factors to analyse women’s leadership across 89 countries in the Olympic movement.

### Democracy, transparency, economic development, and the proportion of women on NOC boards

Findings revealed a positive association between democratic countries and a greater representation of women on NOC boards. Austria, Chile, Mauritius, Portugal, Republic of Korea, Switzerland, and the United Kingdom are among the highly democratic countries where the share of women NOC members ranged from 20 to 29.9%, exceeding the global average (*M* = 19.8). In countries with even higher levels of democracy, such as Australia, Canada, Costa Rica, Estonia, New Zealand, the United States of America, and the Nordic countries (i.e., Denmark, Finland, Iceland, Norway, and Sweden), the rate was above 30%, with some reaching as high as 44.4%. The Nordic countries stood out, with their high levels of freedom, balance between individual and community rights, and well-functioning institutions contributing to the world’s highest rates of women in parliament [3]. Overall, these findings are consistent with previous work in other contexts [20,38,39], suggesting that higher levels of democracy are related to greater visibility of women on NOC boards.

There were however some exceptions to this general pattern. For example, the Czech Republic, Greece, and Uruguay had no women NOC members at all. In Argentina, Bangladesh, Colombia, India, Slovakia, and Thailand, the share of women NOC members was below 10%, with the lowest rate at 3.5%, and a few countries having only one or fewer women on their boards of directors. This was unexpected, given that these countries are considered highly democratic and have more than 20% of women serving in their national parliaments [3], with the exceptions of Colombia, India, and Thailand, where the rate of women legislators ranged from 10 to 15%. The pattern did not hold for these countries due to the prevalence of patriarchal systems in Asian and Latin American cultures, which can perpetuate gender stereotypes and limit women’s opportunities for leadership. Despite the growth of democratic cultures in these countries, traditional gender roles may continue to influence women’s access to leadership.

In contrast, countries with lower levels of democracy, such as Angola, China, Fiji, Rwanda, and Swaziland, had a surprisingly high percentage of women on boards, ranging from 25.8% to 35.7%. One possible explanation may be that region-specific characteristics may also influence the share of women NOC members. Indeed, the Asian region exhibited a weak negative correlation between democracy and the share of women on NOC boards, while the Pacific region showed a weak positive correlation. This highlights the importance of considering regional variability when assessing this relationship. Hence, caution is needed when drawing conclusions from these cases, and the assessments should be refined by examining sub-regional differences within each of these countries.

Governance quality, as measured by transparency, aligns with higher female representation in several cases. Denmark, New Zealand, Singapore, and Sweden, which all scored above 9.1 on the transparency index, had more than 27% women on NOC boards. This indicates that transparent and accountable institutional environments may provide opportunities for women to access leadership positions. Similarly, economic development appears to reinforce this pattern in several countries. For instance, Norway, Switzerland, Sweden, and Denmark, which exhibited relatively high logged GNI per capita scores, also had some of the highest shares of women on NOC boards. In contrast, countries like Fiji, Angola, Romania, and Peru showed relatively strong female representation despite lower GNI per capita scores, suggesting that economic development alone does not fully explain women’s leadership representation. Taken together, democracy, transparency, and economic development appear to create complementary conditions that enhance women’s access to leadership roles.

### Religion and the proportion of women on NOC boards

Consistent with H2 but partially inconsistent with H3a–e, the analysis of 89 countries revealed that religion had a selective influence on the proportion of women NOC members. Countries with a higher share of Protestants were positively associated with women’s representation on NOC boards (*r* = .57, *p* < .01). Surprisingly, and contrary to initial expectations (H3d), countries with a high percentage of Jewish adherents were also positively associated with a greater proportion of women on NOC boards. The other religious traditions did not show significant effects, providing partial support for H3a–c and H3e. Protestant-dominated countries, such as Denmark, Finland, Iceland, Norway, and Sweden stood out, with at least 35% of board positions held by women. Other countries with notable Protestant rates, including Fiji, Germany, Guatemala, Jamaica, Latvia, Malawi, Papua New Guinea, and the United States of America, also had a significant proportion of women on NOC boards (above 20% to 30%). These findings suggest that Protestantism may have a unique influence on women’s representation in leadership positions.

Interestingly, countries with substantial Jewish populations—despite being relatively few in number—also exhibited higher representation of women on NOC boards (e.g., Latvia, Australia, Sweden, Canada, and the United States of America). This unexpected finding suggests that certain religious minority contexts may foster inclusive norms or cultural conditions conducive to gender equity, even in countries where Protestants or other groups are not dominant.

For other religions, although Protestants were not the majority in some Catholic-oriented or religiously diverse countries, such as Angola, Canada, Germany, Guatemala, Lesotho, Rwanda, and Switzerland, our analysis indicated that a higher percentage of Protestants in these countries was still related to a greater proportion of women NOC members. This finding is aligned with existing scholarship [14,41–43], showing that religion plays a role in shaping cultural and political norms in a country. Importantly, our results mirrored empirical research in other areas [20,46], suggesting that countries with a higher share of Protestants are related to a greater representation of women on NOC boards.

Given that the data did not support the link between other religions and gender equality in leadership, as Inglehart and colleagues [20] highlighted, the findings may imply that religious doctrines and values of Protestantism are less restrictive in terms of traditional gender roles and hierarchical structures, promoting gender equity and inclusion in leadership. However, this finding is not universally applicable, as evidenced by Cameroon and Honduras, where the rate of Protestants was above 20%, but the rate of women NOC members was less than 10%. Again, this implies that even in countries with a significant Protestant presence, there may be limited opportunities for women to undertake leadership roles, with an average board size (*M* = 14.7, *SD* = 6.7), resulting in only 1 or 2 women on the board.

Other contrasting patterns included Australia, China, Moldova, Romania, Singapore, and Turkey, where there were fewer than 10% of Protestants, but their share of women NOC members exceeded 25%. In fact, in Australia, Catholics and non-religious individuals made up a larger proportion of the population than that of Protestants. In Singapore, Buddhists outnumbered Protestants, while in Turkey, Muslims dominated. In China and Moldova, the non-religious population was the largest and was higher than that of Protestants. One possible explanation for these cases is that religious freedom and pluralism flourished in Asian and eastern European countries in the late 1980s, despite a long history of religion in these regions. Taken together, the same caution is called when assessing information from these countries since a high share of women NOC members may emerge likely due to broader governance, cultural, and socioeconomic contexts. Therefore, researchers need to carry out further studies to tease out the contextual influences at play.

## Implications, limitations, and future research directions

### Practical and theoretical implications

From a practical standpoint, it is important to note that democratic principles, such as accountability, transparency, free and fair elections, and decentralization of power [13], can be applied at various levels, including continental and international governing bodies. Moreover, promoting diversity, equity, and inclusion on boards is a crucial requirement for democratic procedures [16]. From the critical mass standpoint, having a sufficient number of women on boards is vital to exert substantial influence on decision-making and policy outcomes. In addition, countries with higher levels of economic development are more likely to provide educational and professional opportunities that facilitate women’s advancement into leadership roles, complementing the effects of democratic and transparent institutions [17,20].

While some authoritarian regimes may have increased the number of women leaders in recent years, it is critical to note that these regimes often lack primary democratic principles, leading to limited or weak policy changes. Instead, women leaders may be granted symbolic visibility [16]. In contrast, democratic institutions, coupled with high transparency and robust economic development, offer a framework for fostering gender balance on boards in a more meaningful and effective manner, ultimately enhancing organisational legitimacy [58]. Hence, policymakers are encouraged to adopt democratic processes as a powerful tool for achieving gender balance on boards in their entities.

Furthermore, the findings showed that countries with a high rate of Protestants and Jewish adherents are likely to appoint more women to boards or elect them as board members within their national governing bodies. Possible explanations could be that Protestants are likely to act against social injustice, intolerance, and inequality [48]. Also, the trend observed here supports the contention of Setzler [47] that countries with a larger proportion of Protestants are less likely to emphasise traditional gender roles and hierarchical power structures.

However, we preclude definite conclusions that the tenets and practices of the Protestant faith or Judaism are the sole factors contributing to greater women’s representation in leadership. Indeed, the positive outcomes were often observed in countries with both higher transparency scores (e.g., Denmark, New Zealand, Singapore, Sweden) and higher economic development (e.g., Norway, Switzerland, Sweden, Denmark), suggesting that governance quality and socioeconomic prosperity interact with religious context to support women’s leadership. As shown in some instances, countries with either high rates of other religious adherents and non-religious citizens, or a low share of Protestants or Jewish adherents, have also engaged in efforts to increase women in board and executive positions. Also, we acknowledge that we did not include all other religions, nor did we account for the nuances within the Christian category, such as Orthodox Christians, for example. With these caveats in mind, we recommend that policymakers should not take Protestantism or Judaism as a mark of superiority over other religions, but instead, prioritise religious freedom, pluralism, good governance, economic opportunity, and democratic functioning [42]. By doing so, policymakers can promote board diversity and avoid missing out stakeholders from certain religious or non-religious groups.

When examining board dynamics, professionals and policymakers should consider both the share of boards and board size with caution. For instance, we acknowledge that Papua New Guinea showed the highest rate of women NOC board members (50%), with a board size of 10. This indicates that there were 5 women on NOC boards, but in reality, this case is lamentably rare. In contrast, the United Kingdom had the same number of women NOC members, but their board consisted of 20 members, yielding 25% of women on NOC boards. Hence, policymakers should consider both the number and share of women in leadership positions within organisations. These figures underscore that institutional and structural characteristics, including the size and composition of boards, as well as the broader economic and governance context, matter when evaluating gender representation.

From a theoretical point of view, researchers emphasised the importance of institutional and sociocultural factors in explaining the presence and absence of women in leadership positions across the globe [2,15]. Given the scarcity of research on democracy, governance quality, economic development, religion, and gender imbalance in organisations, this study provides cross-national evidence from 89 countries and advances our understanding of the general associations of women’s representation on NOC boards with levels of democracy and various religions in a country, respectively.

Along these lines, we found correlations between democracy, transparency, economic development, and the share of women on NOC boards, as well as religion and the share of women on NOC boards. Although these correlations are relatively small and moderate in magnitude, we found that democratic political systems and higher transparency scores were positively related to Protestantism and Judaism, highlighting their contributions to supporting gender equality.

Regarding the controls, we observed correlations between the proportion of women on NOC boards and particular regions. Although examining the varying percentage of women on NOC boards across regions was not the focus of this study, the findings supported Norris and Inglehart’s [15] contention that regional characteristics account for the variation in gender inequality in leadership worldwide. We demonstrated that countries in the Americas and Europe were positively correlated with high levels of democracy, while those in Asia, the Middle East and North Africa, and sub-Saharan Africa had a negative correlation. Similarly, there were correlational relationships between religion and region. Consistent with Minkenberg [59], we found that countries with a large share of Protestants were positively correlated with the Americas and the Pacific regions, but negatively correlated with Asia and the Middle East and North Africa.

In line with this, researchers should consider the interplays among democracy, transparency, economic development, religion, and regional characteristics to provide more nuanced explanations for the varying percentage of women on NOC boards. For example, countries with a large rate of Protestants are known as liberal democracies and are mostly located in Western and Northern Europe, but are rarely mirrored in countries in Central and Eastern Europe and Asia [59]. Instead, countries in Central and Eastern Europe, Latin America, and some parts of South and Middle East Asia are influenced by postcommunist social and political systems or strong religious traditions (e.g., Catholic or Muslim), corresponding to social democratic, semidemocratic, or authoritarian regimes [59,60]. This explains some instances of (sub)regional variation in political systems and religion in the Americas, Asia, and Europe. Therefore, researchers need to further explore these possibilities across all other (sub)regions. By examining these factors together, we can gain a better understanding of how they influence women’s representation in leadership positions.

### Limitations and future research directions

There are also some limitations that can inform future studies. First, we used the EIU’s [35] comprehensive democracy barometer, which is a composite score that may not capture all aspects of democracy (e.g., liberal democracy, participatory democracy, and social democracy). From a different lens, researchers have applied other indices, such as the WGI or Polity index to measure democracy and have reiterated that separate indices may require operationalization to represent theoretical foundations [61,62]. Researchers could benefit from employing a broader range of indices to capture the multifaceted nature of democracy more holistically.

Another limitation is that we conducted cross-national comparisons without accounting for critical organisational- and country-level factors that could influence gender dynamics. For example, factors, such as electoral, presidential, and parliamentary systems might be important in understanding gender equality in leadership. These electoral systems are vital for understanding the complexities of gender representation and could yield different narratives if examined in conjunction with our findings. Similarly, we did not examine individual-level variances, as we did not include micro-level factors in our analysis. Technically, the datasets employed in the study entail individual responses that were nested within countries, suggesting the importance of examining micro-level factors to enrich our understanding of gender dynamics in leadership. For instance, variables, such as personal background, education, and socioeconomic status could influence an individual’s likelihood of attaining a leadership position. Hence, our findings should be generalised with caution, and researchers should consider applying multilevel mixed models with these hierarchically structured data or using both micro- and macro-level factors to simultaneously examine gender equality in leadership across countries.

Lastly, it is important to note that this study was cross-sectional, meaning that the interplay among economic development, democracy, religion, and women’s representation on NOCs was examined at a single point in time. Given that cultural and political conditions within each country have evolved, the findings from this study do not capture the long-term effects of these institutional factors. Therefore, researchers should consider utilising longitudinal data that include multiple waves of GNI/GDP per capita, religion, and democracy indices over time to further explore gender dynamics in leadership.

The number and proportion of women in leadership positions have also changed over time. Although social structures, values, and beliefs tend to evolve slowly [14], it would be advantageous to employ panel data that can explain the cross-national variation in the representation of women on NOC boards over a more extended period. In pursuing this line of inquiry, researchers could address critical questions: How are changes in political regimes over time within a country associated with women’s leadership representation in governing bodies? To what extent are changes in women’s leadership representation on NOC boards influenced by a combination of socioeconomic, cultural, and political factors over time?

Overall, conducting an in-depth analysis of time-series data and including explanatory factors at various levels would yield more sophisticated implications for policymakers and academics. By examining the long-term trends and underlying factors affecting women’s representation in leadership roles, researchers can develop a clearer understanding of gender dynamics, ultimately contributing to more effective strategies for promoting gender equality across different sectors.

## Supporting information

S1 File Data. (XLSX)
